# Kinesiophobia Severity Categories and Clinically Meaningful Symptom Change in Persons With Achilles Tendinopathy in a Cross-Sectional Study: Implications for Assessment and Willingness to Exercise

**DOI:** 10.3389/fpain.2021.739051

**Published:** 2021-09-01

**Authors:** Ruth L. Chimenti, Andrew A. Post, Karin Grävare Silbernagel, Katherine Hadlandsmyth, Kathleen A. Sluka, G. Lorimer Moseley, Ebonie Rio

**Affiliations:** ^1^Department of Physical Therapy and Rehabilitation Science, University of Iowa, Iowa City, IA, United States; ^2^Department of Physical Therapy, University of Delaware, Newark, DE, United States; ^3^Department of Anesthesia, Carver College of Medicine, University of Iowa, Iowa City, IA, United States; ^4^Sansom Institute for Health Research, IIMPACT in Health, University of South Australia, Adelaide, SA, Australia; ^5^School of Allied Health, LaTrobe Sport and Exercise Medicine Research Center, La Trobe University, Bundoora, VIC, Australia

**Keywords:** chronic pain, minimal clinically importance difference, tendinopathy, catastrophizing, fear of movement

## Abstract

**Objectives:** (1) Validate thresholds for minimal, low, moderate, and high fear of movement on the 11-item Tampa Scale of Kinesiophobia (TSK-11), and (2) Establish a patient-driven minimal clinically important difference (MCID) for Achilles tendinopathy (AT) symptoms of pain with heel raises and tendon stiffness.

**Methods:** Four hundred and forty-two adults with chronic AT responded to an online survey, including psychosocial questionnaires and symptom-related questions (severity and willingness to complete heel raises and hops). Kinesiophobia subgroups (Minimal ≤ 22, Low 23–28, Moderate 29–35, High ≥ 36 scores on the TSK-11), pain MCID subgroups (10-, 20-, 30-, >30-points on a 0- to 100-point scale), and stiffness MCID subgroups (5, 10, 20, >20 min) were described as median [interquartile range] and compared using non-parametric statistics.

**Results:** Subgroups with higher kinesiophobia reported were less likely to complete three heel raises (Minimal = 93%, Low = 74%, Moderate = 58%, High = 24%). Higher kinesiophobia was associated with higher expected pain (Minimal = 20.0 [9.3–40.0], Low = 43.0 [20.0–60.0], Moderate = 50.0 [24.0–64.0], High = 60.5 [41.3–71.0]) yet not with movement-evoked pain (Minimal = 25.0 [5.0–43.0], Low = 31.0 [18.0–59.0], Moderate = 35.0 [20.0–60.0], High = 43.0 [24.0–65.3]). The most common pain MCID was 10 points (39% of respondents). Half of respondents considered a 5-min (35% of sample) or 10-min (16%) decrease in morning stiffness as clinically meaningful.

**Conclusions:** Convergent validity of TSK-11 thresholds was supported by association with pain catastrophizing, severity of expected pain with movement, and willingness to complete tendon loading exercises. Most participants indicated that reducing their pain severity to the mild range would be clinically meaningful.

## Introduction

Kinesiophobia is defined as fear of movement and re-injury and may interfere with non-operative care for musculoskeletal pain including participation in exercise programs. Additionally, kinesiophobia is associated with disability for a wide range of musculoskeletal pain conditions (1–5). The original 17-item Tampa Scale of Kinesiophobia (TSK-17) was initially validated by demonstrating that individuals with elevated kinesiophobia performed a shorter duration of a lifting task that was maintained until “pain or discomfort made it impossible for the patient to continue” when compared to a group without elevated kinesiophobia (6). To improve feasibility of clinical implementation, the 11-item Tampa Scale of Kinesiophobia (TSK-11) was developed by removing six items from the TSK-17 that had poor psychometric performance (7). Although the TSK-11 is widely used due to the advantage of brevity, it does not yet have well-established thresholds to guide interpretation (8). Therefore, validation is needed to determine if TSK-11 scores are associated with task performance, similar to the original TSK-17.

While the initial studies validating the TSK-17 were in the low back pain population (6, 7), elevated levels of kinesiophobia have also been shown to impact physical function for lower extremity conditions (3–5). For example, individuals with AT and higher levels of kinesiophobia have demonstrated less recovery of calf muscle endurance with an exercise program than participants with lower kinesiophobia (1). Elevated kinesiophobia may contribute to reduced participation and adherence to an exercise program, particularly for a diagnosis like AT where movement-evoked pain is a key diagnostic criteria and exercise is the first line of care. Therefore, clinical assessment of kinesiophobia for those with AT may be warranted. Studies in other chronic pain conditions have indicated that the TSK-11 is positively correlated with pain catastrophizing and pain severity (9–12). Validation of the TSK-11 for patients with AT would be strengthened by examining the convergent validity of the TSK-11 with pain catastrophizing, symptom severity, and willingness to complete tendon loading activities used in home exercise programs.

In addition to screening for relevant pain-related psychological factors, determining a patient's target level of symptoms (e.g., pain, stiffness) can assist with development of collaborative and patient-centered goals. Movement-evoked pain and stiffness at rest are key symptoms of AT, yet they lack formally validated minimal clinically important differences (MCIDs) in the literature. For pain in general, a 30% decrease was best associated with a patient global rating of change of “much improved or better” among 2,724 patients with a variety of musculoskeletal pain conditions participating in multi-center pain studies (13). A 30% reduction in pain is also consistent with the MCID criteria proposed by the OMERACT study group (Outcome Measurement in Rheumatoid Arthritis Clinical Trial) (14). Although duration of morning stiffness is the first question on the Victorian Institute of Sport-Achilles (VISA-A) questionnaire (15), no MCID has been established for this symptom. Further research is needed to determine if a 30% decrease is an appropriate MCID for movement-evoked pain and stiffness and to evaluate patient-defined acceptable levels for AT symptoms.

The first purpose of this study was to validate four categories of minimal, low, moderate, and high fear of movement/re-injury on the TSK-11. We hypothesized that convergent validity of the TSK-11 subgroups would be supported by groups of higher kinesiophobia reporting higher pain catastrophizing, higher AT symptom severity, and lower willingness to complete Achilles tendon loading activities. The second purpose was to develop a patient-driven MCID for AT symptoms of pain with heel raises and stiffness at rest. Given that previous studies have reported an association between fear of movement, pain catastrophizing, and pain severity (9–12), a secondary purpose was to determine whether fear of movement or pain catastrophizing were associated with the magnitude of the respondent's chosen MCID for pain and stiffness.

## Materials and Methods

**Figure 1:**
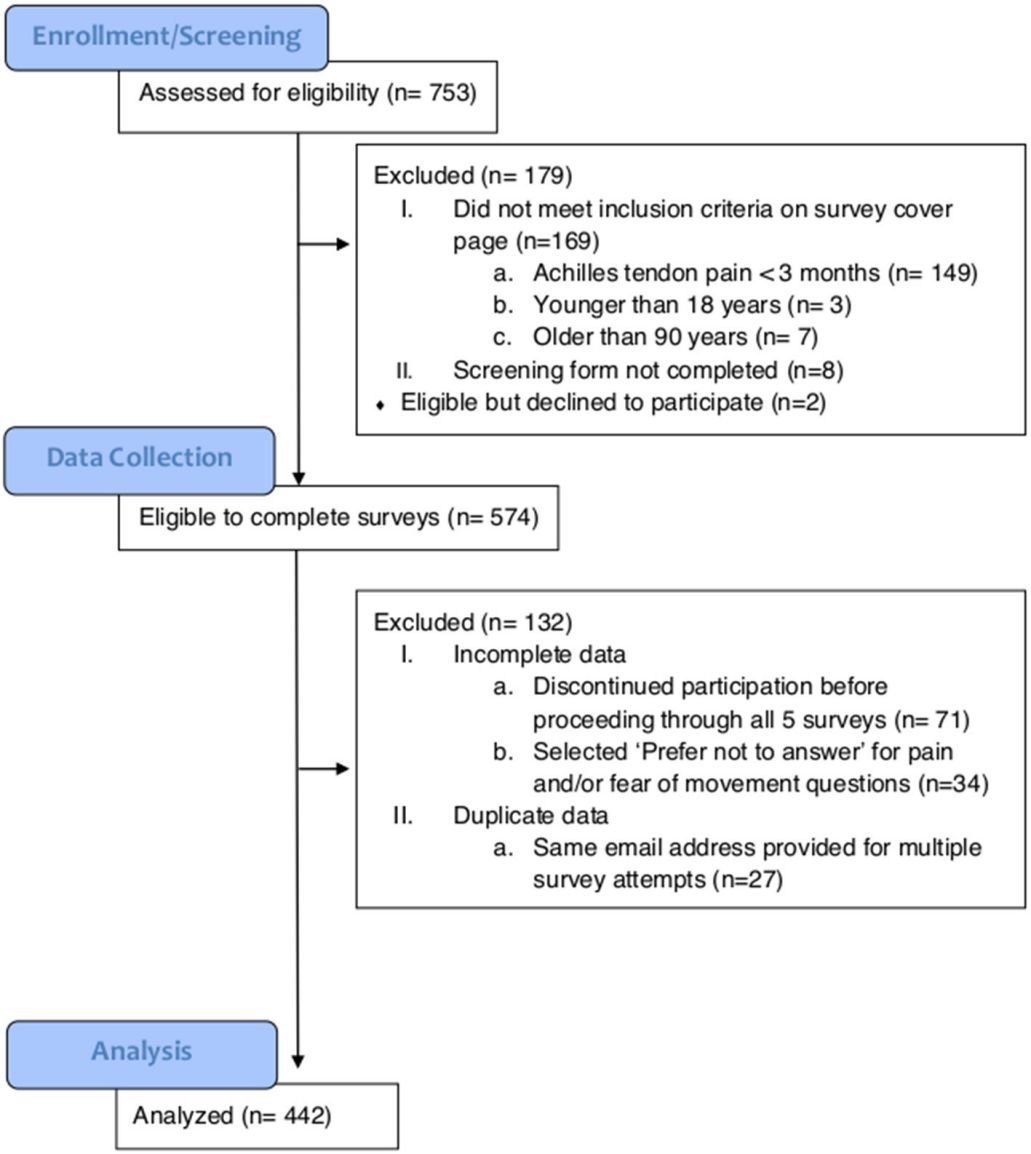
CONSORT diagram indicating the number of individuals at enrollment, data collection, and analysis.

For this cross-sectional study, a public online survey link was emailed to individuals who had previously participated in research studies on AT at three universities and was posted on a website that provided educational resources about pain to the public (bodyinmind.org). From November 2018 through May 2019, 753 people completed an online screening form with the inclusion criteria of (1) self-reported Achilles tendon pain >3 months, and (2) between 18 and 90 years of age (Figure 1). A total of 574 survey responders met the inclusion criteria and proceeded to the five self-reported outcomes: (1) Demographics, (2) Fear of Movement, (3) Pain Catastrophizing, (4) Pain description, and (5) Fibromyalgia severity (FS) scale. Respondents were excluded if the data were incomplete (ended participation before proceeding through all five surveys, *n* = 71; selected “Prefer not to answer” for pain or fear of movement questions, *n* = 34) or the same email address provided for multiple responses (*n* = 27), resulting in final analysis of *N* = 442 responses. Potential participants were provided with the elements of consent prior to proceeding to the survey questions. Respondents who chose to provide their email address were sent a $5 gift ecard. The funder played no role in the design, conduct, or reporting of this study. The study protocol and analysis is consistent with the purposes provided to potential respondents on the screening page.

### Psychosocial Questionnaires

Abbreviated versions of the TSK-11 and the Pain Catastrophizing Scale (PCS-4), as well as one-question fear of movement and pain catastrophizing screens, were used to assess comparability of these pain-related psychological factors. Fear of movement was captured with the TSK-11 that has participants rate their agreement with 11 statements that indicate fear of movement/re-injury on a 4-point Likert scale from (1 = “Strongly disagree” to 4 = “Strongly agree,” score range 11–44) (7). The one-question kinesiophobia screen was: “Physical activity might damage me” on a 101-point scale from 0, “Completely disagree,” to 100, “Completely agree” (16). Pain catastrophizing was captured with the PCS-4 that captures the degree of catastrophizing thoughts when in pain on a 5-point Likert scale (0 = “Not at all” to 4 = “All the time,” score range 0–16) (17). The one-question pain catastrophizing screen was: “When I feel the pain, it is terrible and I feel that it's never going to get better” on a 101-point scale from 0, “Completely disagree,” to 100, “Completely agree”) (16).

### Determination of TSK-11 Thresholds

Thresholds for the TSK-11 subgroups align with previously established quartiles in a large sample of patients with variety of chronic pain conditions (18). The use of previously published data with a normal distribution facilitates interpretation of the level of kinesiophobia as minimal, low, moderate, or high (18). To convert the TSK-17 thresholds (25^th^ percentile = 35, mean = 41, 75^th^ percentile = 48) thresholds to TSK-11 thresholds, the score was based on the minimum number of items needed with an “Agree” response for Low and Moderate thresholds and “Strongly Agree” for the High threshold (see Supplementary Table 1 for an excel document with calculations). For example, the threshold for Low kinesiophobia (TSK-11 = 23, TSK-17 = 35) is reached when a participant responds affirmatively (score ≥3) to at least one-question with all other questions scored at 2 (each question is scored on 4-point scale from 1 to 4). The threshold for Moderate kinesiophobia (TSK-11 = 29, TSK-17 = 41) is reached by selecting “Agree” for at least seven questions and marking the remaining questions as “Disagree.” The threshold for High kinesiophobia (TSK-11 = 36, TSK-17 = 48) is reached when at least three questions are scored at 4 (strongly agree), eight questions are scored at 3 (agree), and remaining questions are scored at 2 (disagree).

### Symptom-Related Questions (Severity and Willingness to Complete Activities)

**Figure 2:**
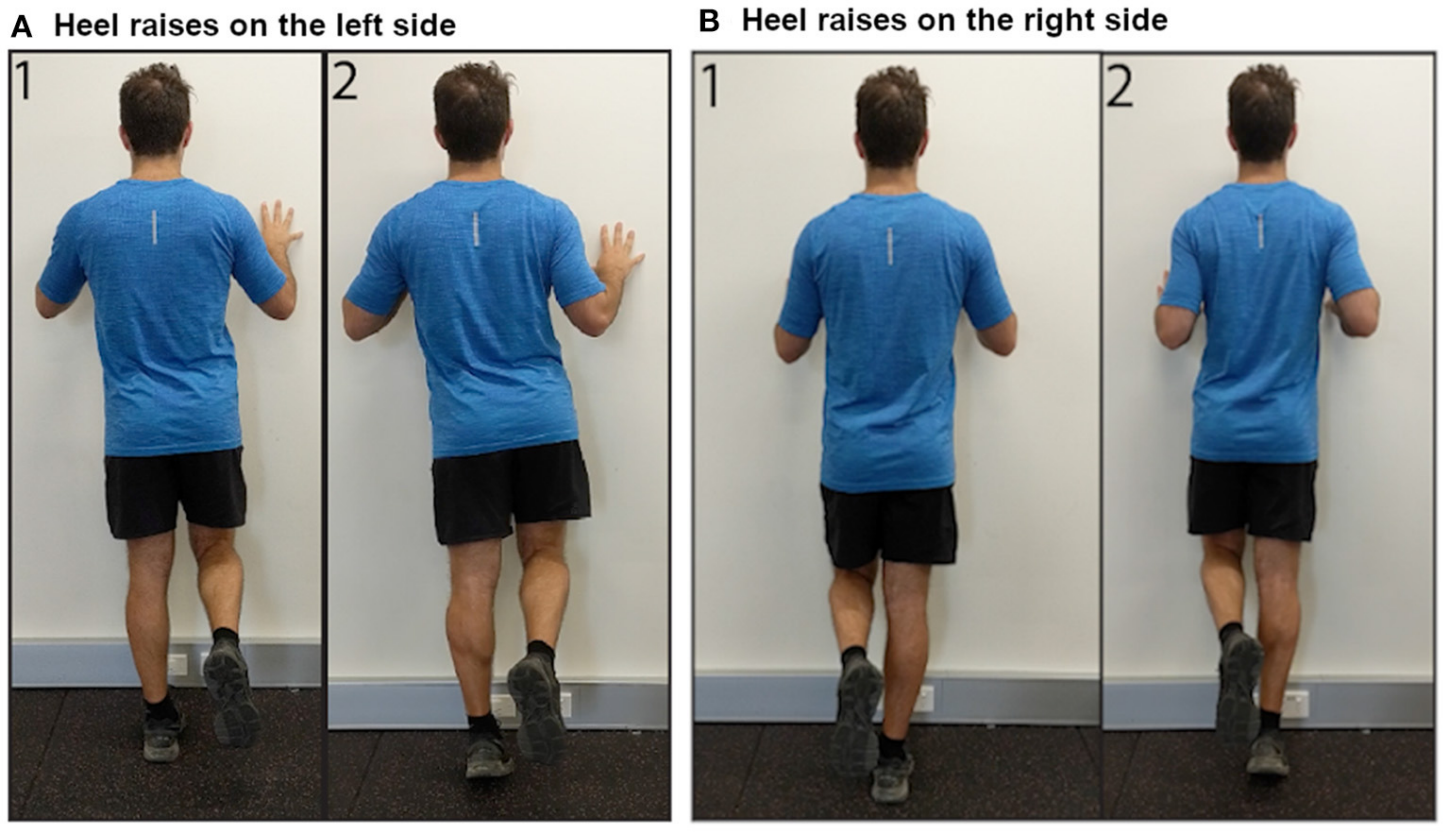
Images demonstrating performance of single leg heel raises on the **(A)** left side, and **(B)** right side.

**Figure 3:**
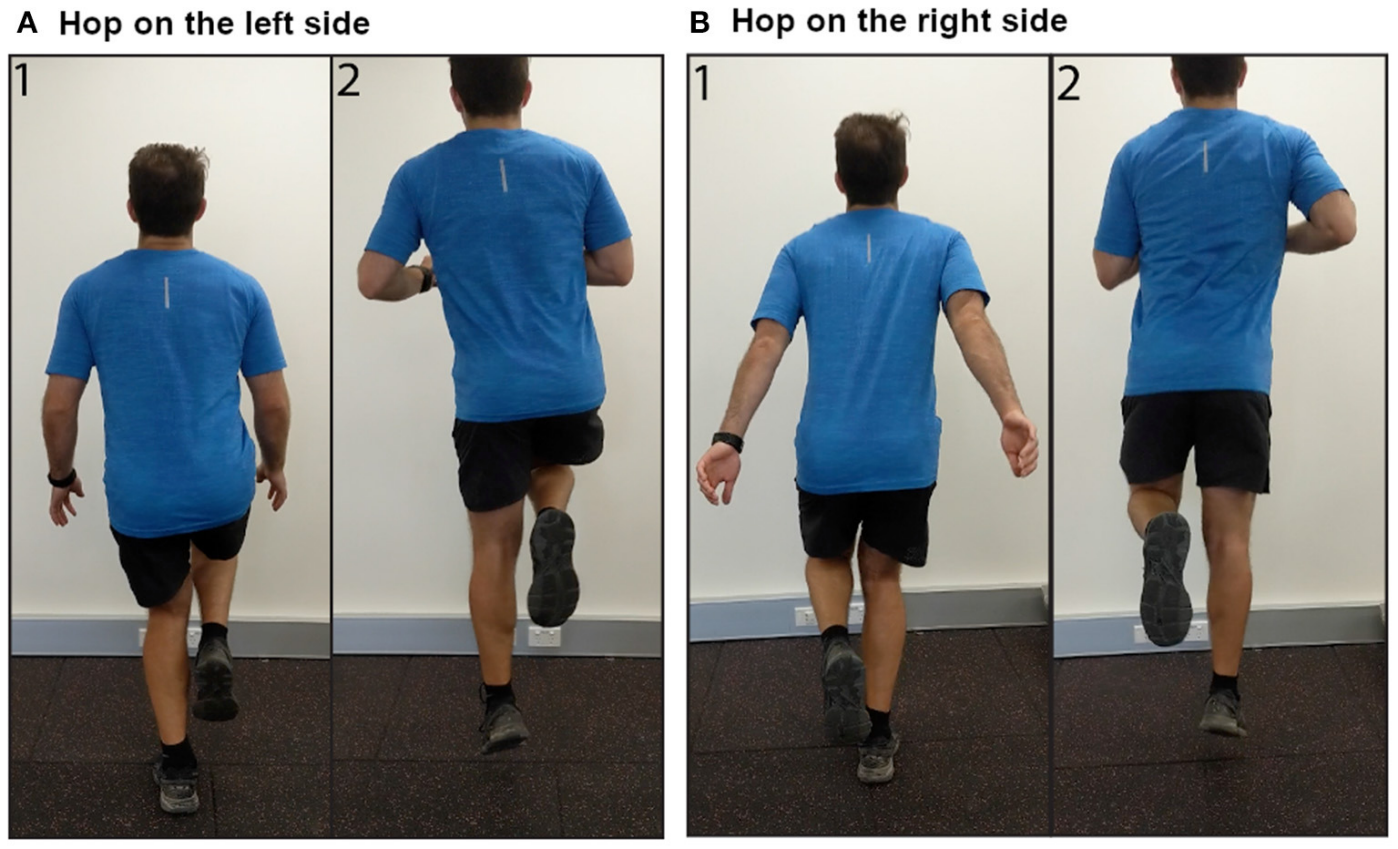
Images demonstrating performance of single leg hops on the **(A)** left side, and **(B)** right side.

Respondents provided information on laterality of AT (unilateral or bilateral). Those with bilateral AT also reported their more painful side (left or right). Respondents were asked to rate their AT pain when sitting in a chair at rest [Visual Analog Scale (VAS) from 0, “No pain,” to 100, “Pain as bad as you can imagine”]. Respondents were then given the instructions, “See the following two images demonstrating a heel raise OR download the following video demonstration” (Figure 2, Videos for left and right sides available as Supplementary Videos 1, 2). The side shown in the image and video corresponded to which side (left or right) the respondent reported as more painful. Respondents reported their expected pain (VAS) during three single leg heel raises on their more painful side and whether they were willing to complete the activity. Respondents who were willing to complete three single-leg heel raises rated their movement-evoked pain (VAS) after completing the heel raises. Respondents were then given the instructions, “See the following two images demonstrating a hop OR download the following video demonstration” (Figure 3, Videos for left and right sides available as Supplementary Videos 3, 4). Respondents reported their expected pain (VAS) during three single leg hops on their more painful side and whether they were willing to complete the activity. Respondents willing to complete hops rated their movement-evoked pain (VAS) after completing the hops. Respondents unwilling to complete the heel raises and/or hops selected their primary rationale (“I am unable to do,” “It would be too painful,” “I am afraid I would hurt myself,” “I am not in a location where I can try this exercise,” “Other”). For morning tendon stiffness, participants were asked, “For how many minutes do you have stiffness in the Achilles tendon region on first getting up?” and could record duration from 0 to ≥ 100 min.

The source of AT symptoms is most commonly peripheral (19, 20), but alterations in how the central nervous system processes nociceptive input could also contribute to an individual's AT symptoms. As defined by the International Association for the Study of Pain (IASP), nociplastic pain “arises from altered nociception despite no clear evidence of actual or threatened tissue damage causing the activation of peripheral nociceptors or evidence for disease or lesion of the somatosensory system causing the pain” (21). To screen for the presence of nociplastic pain, we used the FS scale (22). In any patient with musculoskeletal pain, the total severity of symptoms is the sum of two parts: (1) presence of pain in 19 body areas (Widespread Pain Index: score 0–19) (23), and (2) the Symptoms Severity Scale (Sum of fatigue, waking unrefreshed, cognitive symptoms, headaches, pain/cramps in abdomen, depression, headache: score 0–12) (22). A total score ≥13 has been used as a threshold to identify people for further evaluation of fibromyalgia symptoms (22, 23).

### Determination of Minimal Clinically Important Difference Thresholds

**Figure 4:**
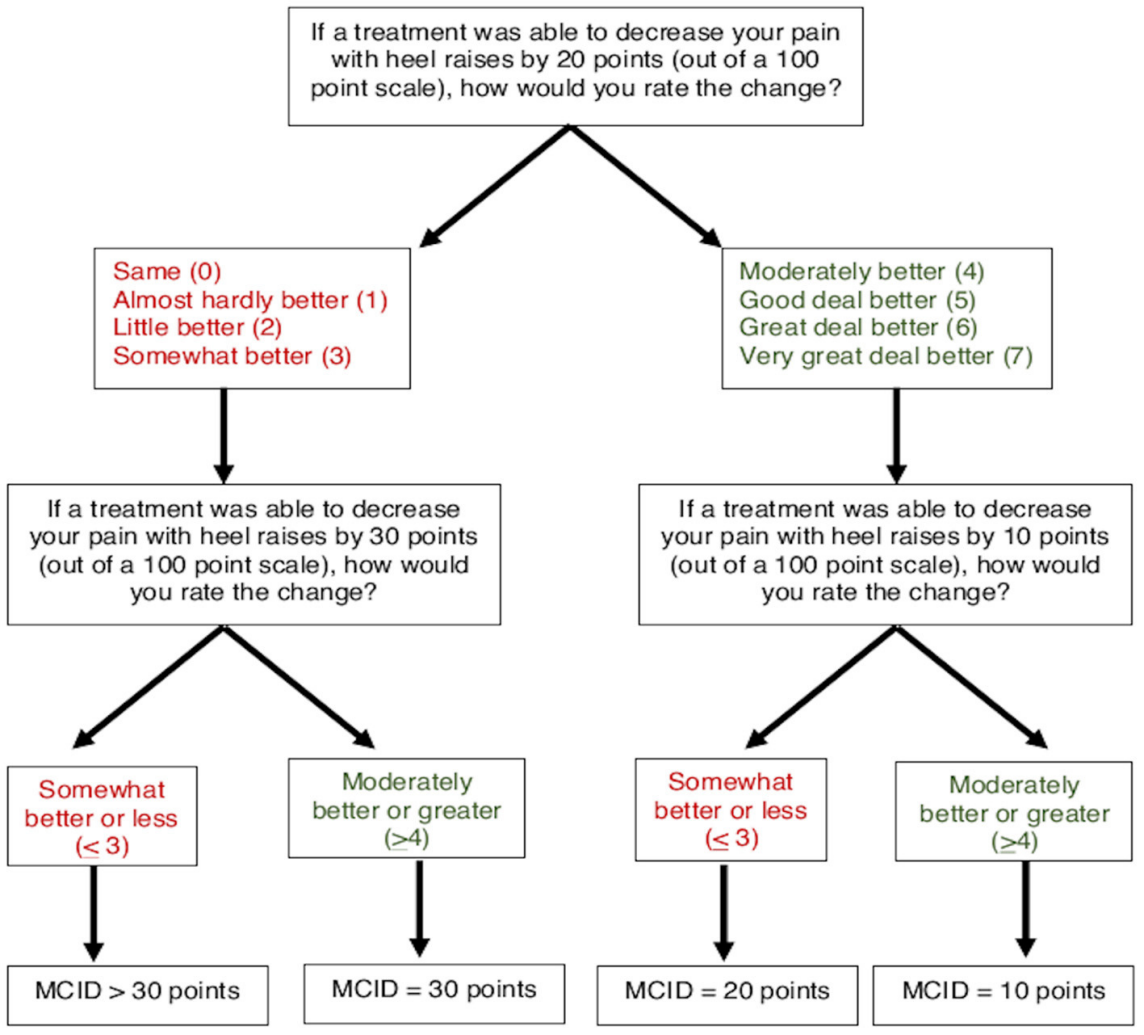
Flow chart for determining patient-driven MCID for pain with heel raises.

**Figure 5:**
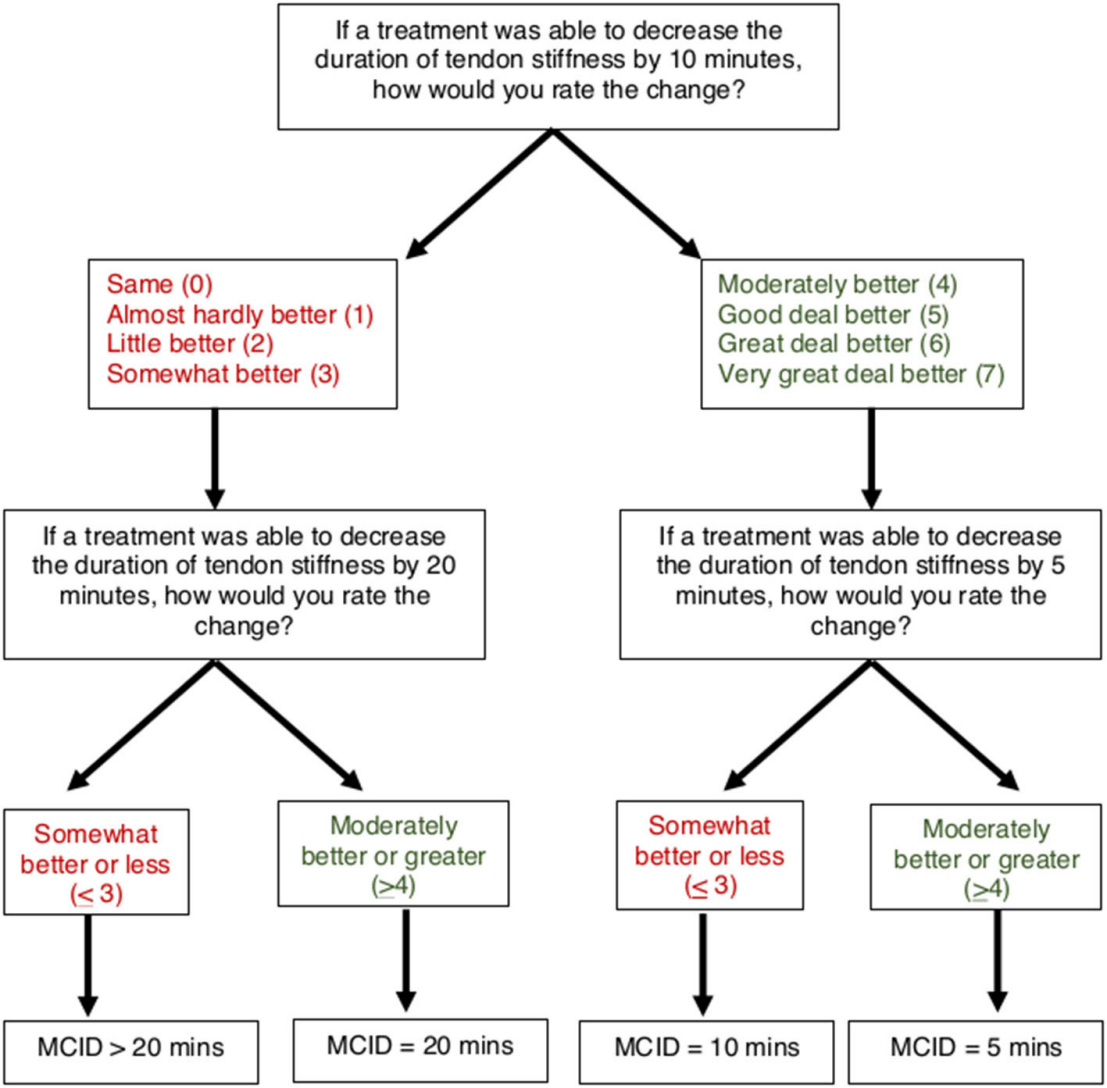
Flow chart for determining patient-driven MCID for Achilles tendon stiffness.

To determine MCIDs for AT symptoms of pain and stiffness, an anchor-based approach was used to match the magnitude of anticipated change in a patient-reported outcome (VAS for pain and stiffness) with a Global Rating of Change (7-point scale). A threshold of “Moderately better” or greater (≥4) was used to indicate the MCID for pain and stiffness, which has been previously used to define a clinically meaningful improvement for AT symptoms (24). A smart-form that increased (or decreased) the potential change in symptoms was used to determine the respondents' MCID (Figures 4, 5). For pain, participants were asked, “If a treatment was able to decrease your pain with heel raises by 20 points (on a 0–100 scale), how would you rate this change?” on a 7-point scale from 0 (“Same”) to 7 (“Very great deal better”). Participants who rated a 20-point decrease as “Moderately better” or greater (≥4) were subsequently asked about perceived magnitude of change for smaller 10-point decrease. Participants who rated a 20-point decrease as “Somewhat better” or less (≤ 3) were subsequently asked about magnitude of change for a larger 30-point decrease. Participants who ranked a 30-point decrease in pain as “Somewhat better” or less (≤ 3) were categorized as MCID >30 points. Similarly, for stiffness participants were asked, “If a treatment was able to decrease the duration of tendon stiffness by 10 min, how would you rate this change?” on a 7-point scale from 0 (“Same”) to 7 (“Very great deal better”). Participants who rated a 10-min decrease as “Moderately better” or greater (>4) were subsequently asked about perceived magnitude of change for a smaller 5-min decrease. Participants who rated a 10-min decrease as “Somewhat better” or less (≤ 3) were subsequently asked about magnitude of change for a larger 20-min decrease. Participants who ranked a 20-min decrease in stiffness as “Somewhat better” or less (≤ 3) were categorized as MCID >20 min.

Four subgroups based on respondent-chosen MCID were defined for AT symptoms of pain (decrease of 10, 20, 30, or >30 points) and duration of morning tendon stiffness (decrease of 5, 10, 20, or >20 min). Target pain intensity with heel raises was calculated as the expected pain with heel raises (VAS, 0–100) minus the pain MCID. Target percent decrease in tendon pain with heel raises was calculated as the pain MCID divided by the expected pain with heel raises multiplied by 100.


$$\begin{array}{l} \text{ Target pain intensity}=\;\left(\left[\text{Expected pain with heel }raises\right]\;-\;\left[Pain\;MCID\right]\right)\;\\ \text{Target percent decrease in pain }=\;{\left(\left[\text{Target pain intensity}\right]\;/\;\left[Pain\;MCID\right]\right)}^{*}\;100 \end{array}$$

Target pain intensity and target percent decrease in pain are only for the heel raise task because the pain MCID was specific to heel raises. Expected pain with heel raises was used for the calculation of target pain intensity rather than movement-evoked pain during the heel raises due to high percentages of respondents unwilling to complete the task across all groups. Target stiffness duration was calculated as the duration of tendon stiffness minus the smallest chosen MCID. Target percent decrease in tendon stiffness was calculated as the stiffness MCID divided by reported duration of tendon stiffness multiplied by 100. Because the >30-point pain MCID and >20-min stiffness MCID subgroups were not offered an option large enough to be considered clinically meaningful, target pain intensity/stiffness duration and percent decrease were not calculated.

### Analysis

Convergent validity of the TSK-11 subgroups with levels of pain catastrophizing and severity of AT symptoms were examined using parametric and non-parametric statistics, as appropriate. Continuous variables were checked for normality with Shapiro-Wilk tests and the Normal Q-Q plot. Continuous variables that were not normally distributed were described using median [interquartile range] and were compared between groups using non-parametric statistics. Kruskal Wallis tests were used to compare continuous variables between subgroups for the TSK-11 and MCIDs for pain and stiffness. Mann-Whitney tests were used for *post-hoc* between-group comparisons. Dichotomous variables were compared between groups using Chi-Square Pearson tests. Convergent validity of the TSK-11 subgroups with willingness to complete tendon loading activities was examined using a Receiver Operating Curve (ROC) analysis. The area under the curve was used to examine how well the TSK-11 was able to predict willingness to complete activities. The sensitivity and specificity values for each threshold were also examined. Statistical significance was determined by a two-tailed alpha of 0.05. To control for multiple between-group comparisons, the *p*-value for statistical significance was adjusted for a Bonferroni correction of 20 dependent variables. Pairwise deletion was used to omit missing data from the analysis.

## Results

### Demographics

A total of 442 respondents included individuals from the United States (*n* = 424), Australia (*n* = 14), Germany (*n* = 1), India (*n* = 1), New Zealand (*n* = 1), and United Kingdom (*n* = 1). There were no differences between groups for age, sex, and BMI (Table 1). No respondents in the Minimal-kinesiophobia group identified as Hispanic or Latino, whereas 46.4% of the High-kinesiophobia group identified as Hispanic or Latino (Table 1). The High-kinesiophobia group also had fewer respondents who identified as Caucasian compared to the Minimal-kinesiophobia group (66.8 vs. 93.1%, *p* = 0.009). Most participants sought care from at least two providers and had tried at least three treatments for AT (Table 1).

**Table 1:** Demographics, fear of movement, and pain catastrophizing compared between groups by levels of kinesiophobia on the 11-item Tampa Scale of Kinesiophobia (TSK-11) score as Minimal (TSK ≤ 22), Low (TSK 23–28), Moderate (TSK 29–35), and High (TSK ≥36).

	**Minimal**	**Low**	**Moderate**	**High**	***p*-Value**
	***n* = 44**	***n* = 81**	***n =* 121**	***n* = 196**	
**DEMOGRAPHICS**
**Age** (years)	37.0 [30.0–51.0]	36.5 [25.5–46.0]	36.4 [29.3–42.0]	36.0 [30.0–40.0]	1.0
**Sex** (#, % Women)	31/44 (70.5%)	53/81 (65.4%)	76/121 (62.8%)	91/196 (46.4%)	**0.020**
*post-hoc P*-value	1.0^a^	1.0^b^	0.100^c^	0.080^d^	0.080^e^
**BMI**	25.5 [22.8–30.6]	24.6 [22.2–27.5]	24.7 [23.1–26.2]	25.4 [23.7–27.8]	1.0
**Hispanic or Latino** (% Yes)	0/44 (0%)	19/81 (23.5%)	28/121 (23.1%)	91/196 (46.4%)	** <0.001**
*post-hoc P*-value	**0.011** ^a^	1.0^b^	**0.002** ^c^	**0.008** ^d^	** <0.001** ^e^
**Race (% yes, not mutually exclusive)**				
Caucasian	41/44 (93.1%)	61/81 (75.0%)	79/117 (67.5%)	131/196 (66.8%)	**0.040**
*post-hoc P*-value	0.280^a^	1.0^b^	1.0^c^	1.0^d^	**0.009** ^e^
Black or African American	1/44 (2.3%)	4/81 (4.9%)	18/121 (14.9%)	39/196 (19.9%)	**0.020**
*post-hoc P*-value	1.0^a^	0.520^b^	1.0^c^	0.040^d^	0.100^e^
Asian	1/44 (2.3%)	13/81 (16.0%)	10/121 (8.3%)	17/196 (8.7%)	1.0
Other	1/44 (2.3%)	1/81 (1.2%)	11/121 (9.0%)	9/196 (4.6%)	0.778
**Providers seen** (#)	2.0 [1.0–3.0]	2.0 [1.0–3.0]	2.0 [1.0–3.0]	2.0 [1.0–3.0]	1.0
**Treatments tried** (#)	5.0 [4.0–7.0]	4.0 [2.0–6.0]	3.0 [2.0–5.0]	3.0 [2.0–4.0]	** <0.001**
*post-hoc P*-value	1.0^a^	0.660^b^	**0.020** ^c^	** <0.001** ^d^	** <0.001** ^e^
**FEAR OF MOVEMENT/RE-INJURY**
**TSK-11** (11–44)	19.5 [16.8–21.3]	26.0 [25.0–27.0]	33.0 [30.0–35.0]	38.0 [37.0–40.0]	** <0.00**
*post-hoc P*-value	** <0.001** ^**a**^	** <0.001** ^**b**^	** <0.001** ^**c**^	** <0.001** ^d^	** <0.001** ^e^
**One-question screen** (0–100)	2.5 [0.0–12.5]	41.0 [20.0–62.0]	59.0 [36.0–67.5]	64.0 [50.0–74.8]	** <0.001**
*post-hoc P*-value	** <0.001** ^**a**^	**0.020** ^**b**^	**0.007** ^**c**^	** <0.001** ^d^	** <0.001** ^e^
**PAIN CATASTROPHIZING**
**PCS-4** (0–16)	4.0 [3.0–6.8]	6.0 [4.0–9.0]	9.0 [7.0–11.0]	12.0 [10.0–14.0]	** <0.001**
*post-hoc P*-value	**0.028** ^a^	** <0.001** ^b^	** <0.001** ^**c**^	** <0.001** ^d^	** <0.001** ^e^
**One-question screen** (0–100)	9.0 [0.3–29.5]	35.0 [20.0–59.0]	58.0 [37.0–66.0]	65.0 [57.0–74.0]	** <0.001**
*post-hoc P*-value	** <0.001** ^a^	** <0.001** ^b^	** <0.001** ^**c**^	** <0.001** ^d^	** <0.001** ^e^

*The scale range is provided in parentheses following the name of the outcome measure. Continuous variables presented as median [interquartile range] with p-values for Kruskal Wallis test and post-hoc Mann-Whitney U tests. Categorical data presented as % (#/n) with P-values for Pearson Chi-Square tests. PCS-4, 4-item Pain Catastrophizing Scale*.

a *P-value for comparison of TSK ≤ 22 (Minimal) vs. TSK 23–28 (Low)*.

b *P-value for comparison of TSK 23–28 (Low) vs. TSK 29–35 (Moderate)*.

c *P-value for comparison of TSK 29–35 (Moderate) vs. TSK > 36 (High)*.

d *P-value for comparison of TSK 23–28 (Low) vs. TSK ≥ 36 (High)*.

e *P-value for comparison of TSK ≤ 22 (Minimal) vs. TSK ≥ 36 (High)*.

*Statistically significant p-values are in bold*.

### Convergent Validity of the TSK-11 Subgroups

#### Pain Catastrophizing

The TSK-11 scores in this sample were left-skewed with the majority of respondents reporting moderate to high kinesiophobia. Respondents were categorized into four groups based on TSK-11 score: Minimal: *n* = 44 (12.2% of sample TSK ≤ 22/44), Low: *n* = 81 (22.4% 23–28/44), Moderate: *n* = 121 (27.4% 29–35/44), and High: *n* = 196 (44.3% >36). The kinesiophobia groups demonstrated progressively higher scores on the PCS-4 as well as the pain catastrophizing screens on all *post-hoc* tests (Table 1).

#### Severity of Symptoms

Kinesiophobia groups differed in expected pain and behavior yet not in movement-evoked pain during tendon-loading exercises. A higher level of kinesiophobia was associated with a higher expected intensity of pain during activity (*p* < 0.001 for heel raises and hops, Table 2). However, there were no differences in movement-evoked pain during activity between groups (heel raises: *p* = 0.060, hops: *p* = 1.0, Table 2). Between group comparisons of movement-evoked pain during activities should be interpreted with caution given the low rates (25.0–57.9%) of respondents willing to complete heel raises or hops in the Moderate and High kinesiophobia groups (Table 2).

**Table 2:** Achilles tendon pain (VAS, 0–100) and willingness to complete tendon-loading activities compared between groups stratified by levels of kinesiophobia on the TSK-11 score as Minimal (TSK ≤ 22), Low (TSK 23–28), Moderate (TSK 29–35), and High (TSK ≥ 36).

	**Minimal**	**Low**	**Moderate**	**High**	***p*-Value**
	***n* = 44**	***n* = 81**	***n* = 121**	***n* = 196**	
**Pain at rest** (0–100)	5.0 [0.0–12.8]	13.0 [4.5–50.0]	28.5 [8.3–60.5]	61.0 [34.0–73.0]	** <0.001**
*post-hoc P*-value	**0.004** ^a^	0.080^b^	** <0.001** ^c^	** <0.001** ^d^	** <0.001** ^e^
**Duration of tendon stiffness** (min)	15.0 [8.0–30.0]	30.0 [15.8–50.0]	37.0 [21.0–59.0]	58.0 [34.0–66.0]	** <0.001**
*post-hoc P*-value	0.100^a^	1.0^b^	** <0.001** ^c^	** <0.001** ^d^	** <0.001** ^e^
**Expected pain with three heel raises** (0–100)	20.0 [9.3–40.0]	43.0 [20.0–60.0]	50.0 [24.0–64.0]	60.5 [41.3–71.0]	** <0.001**
*post-hoc P*-value	**0.001** ^a^	1.0^b^	** <0.001** ^c^	** <0.001** ^d^	** <0.001** ^e^
Movement-evoked pain with three heel raises^*^ (0–100)	25.0 [5.0–43.0]	31.0 [18.0–59.0]	35.0 [20.0–60.0]	43.0 [24.0–65.3]	0.060
	Missing, *n* = 3	Missing, *n* = 21	Missing, *n* = 51	Missing, *n* = 148	
**Expected pain with three hops** (0–100)	35.0 [15.0–50.0]	50.0 [27.5–64.5]	53.0 [27.0–68.5]	59.0 [34.0–72.0]	** <0.001**
*post-hoc P*-value	**0.001** ^a^	1.0^b^	0.060^c^	0.180^d^	** <0.001** ^e^
Movement-evoked pain with three hops^*^ (0–100)	32.0 [12.0–50.0]	38.5 [21.0–60.0]	39.0 [26.0–61.0]	37.0 [26.0–58.0]	1.0
	Missing, *n* = 6	Missing, *n* = 30	Missing, *n* = 57	Missing, *n* = 147	
**Completed heel raises** (% yes)	41/44 (93.2%)	60/81 (74.1%)	70/121 (57.9%)	48/196 (24.4%)	** <0.001**
*post-hoc P*-value	0.260^a^	1.0^b^	** <0.001** ^c^	** <0.001** ^d^	** <0.001** ^e^
**Completed hops** (% yes)	38/44 (86.4%)	51/81 (63.0%)	64/121 (52.9%)	49/196 (25.0%)	** <0.001**
*post-hoc P*-value	0.200^a^	1.0^b^	** <0.001** ^c^	** <0.001** ^d^	** <0.001** ^e^

*P-values are Bonferroni adjusted for multiple comparisons. Data presented as median [interquartile range] with p-values for Kruskal Wallis tests and post-hoc Mann-Whitney U tests. Categorical data is presented as % (#/n) with p-values for Pearson Chi-Square tests*.

* *Movement-evoked pain during activity only rated by respondents who were willing to do these activities, otherwise data considered missing*.

a *P-value for comparison of TSK ≤ 22 (Minimal) vs. TSK 23–28 (Low)*.

b *P-value for comparison of TSK 23–28 (Low) vs. TSK 29–35 (Moderate)*.

c *P-value for comparison of TSK 29–35 (Moderate) vs. TSK ≥36 (High)*.

d *P-value for comparison of TSK 23–28 (Low) vs. TSK ≥36 (High)*.

e *P-value for comparison of TSK ≤ 22 (Minimal) vs. TSK ≥36 (High)*.

*Statistically significant p-values are in bold*.

Kinesiophobia groups differed in AT symptom measures, including resting pain and stiffness (Table 2). There was a progressive increase in pain at rest between groups with the High kinesiophobia group having a resting pain intensity that was over 12 times greater than the Minimal kinesiophobia (61 vs. 5/100, Table 2). Similarly, the High kinesiophobia group reported a nearly four times longer duration of Achilles tendon stiffness on first getting up than the Minimal kinesiophobia group (58 vs. 15 min, Table 2). Signs of nociplastic pain were low among respondents. Although the High kinesiophobia subgroup reported relatively higher FS score than the other subgroups (Table 3), no respondents had a score ≥13, which is a threshold used to identify people for further evaluation of fibromyalgia symptoms (22, 23).

**Table 3:** Nociplastic pain indicators compared between groups stratified by levels of kinesiophobia on the TSK-11 score as Minimal (TSK ≤ 22), Low (TSK 23–28), Moderate (TSK 29–35), and High (TSK ≥36).

	**Minimal**	**Low**	**Moderate**	**High**	***p*-Value**
	***n* = 44**	***n* = 81**	***n* = 121**	***n* = 196**	
**Fibromyalgia severity score** (0–31)	3.0 [2.0–6.0]	5.0 [4.0–7.0]	5.0 [4.0–8.0]	6.0 [5.0–8.0]	** <0.001**
*post-hoc p*-value	0.020^a^	1.0^b^	1.0^c^	0.020^d^	** <0.001** ^e^
**Widespread pain index** (0–19)	1.0 [0.0–1.0]	1.0 [0.0–2.0]	1.0 [0.0–2.0]	0.0 [0.0–1.0]	1.0
**Symptom severity score** (0–12)	3.0 [1.0–5.0]	4.0 [3.0–6.0]	5.0 [4.0–7.0]	5.5 [4.0–7.8]	** <0.001**
*post-hoc p*-value	**0.006** ^a^	1.0^b^	0.42^c^	** <0.001** ^d^	** <0.001** ^e^
**Severity of fatigue** (0–3)	1.0 [0.0–1.8]	1.0 [1.0–2.0]	1.0 [1.0–2.0]	2.0 [1.0–2.0]	** <0.001**
*post-hoc p*-value	**0.001** ^a^	0.860^b^	0.760^c^	**0.004** ^d^	** <0.001** ^e^
**Severity of waking unrefreshed** (0–3)	1.0 [0.0–1.0]	1.0 [1.0–2.0]	2.0 [1.0–2.0]	2.0 [1.0–2.0]	** <0.001**
*post-hoc p*-value	**0.040** ^a^	1.0^b^	1.0^c^	**0.040** ^d^	** <0.001** ^e^
**Severity of cognitive symptoms** (0–3)	0.0 [0.0–1.0]	1.0 [0.0–1.0]	1.0 [1.0–2.0]	2.0 [1.0–2.0]	** <0.001**
*post-hoc p*-value	0.060^a^	** <0.001** ^b^	**0.004** ^c^	** <0.001** ^d^	** <0.001** ^e^
**Headaches** (0–1)	0.0 [0.0–1.0]	0.0 [0.0–1.0]	0.0 [0.0–1.0]	0.0 [0.0–1.0]	1.0
**Pain or cramps in lower abdomen** (0–1)	0.0 [0.0–1.0]	0.0 [0.0–0.0]	0.0 [0.0–0.0]	0.0 [0.0–0.0]	1.0
**Depression** (0–1)	0.0 [0.0–0.0]	0.0 [0.0–1.0]	0.0 [0.0–0.0]	0.0 [0.0–0.0]	1.0

*P-values are Bonferroni adjusted for multiple comparisons. Data presented as median [interquartile range] with p-value for Kruskal Wallis test and post-hoc Mann-Whitney U tests*.

a *P-value for comparison of TSK ≤ 22 (Minimal) vs. TSK 23–28 (Low)*.

b *P-value for comparison of TSK 23–28 (Low) vs. TSK 29–35 (Moderate)*.

c *P-value for comparison of TSK 29–35 (Moderate) vs. TSK > 36 (High)*.

d *P-value for comparison of TSK 23–28 (Low) vs. TSK ≥ 36 (High)*.

e *P-value for comparison of TSK ≤ 22 (Minimal) vs. TSK ≥ 36 (High)*.

*Statistically significant p-values are in bold*.

#### Willingness to Complete Tendon Loading Activities

**Figure 6:**
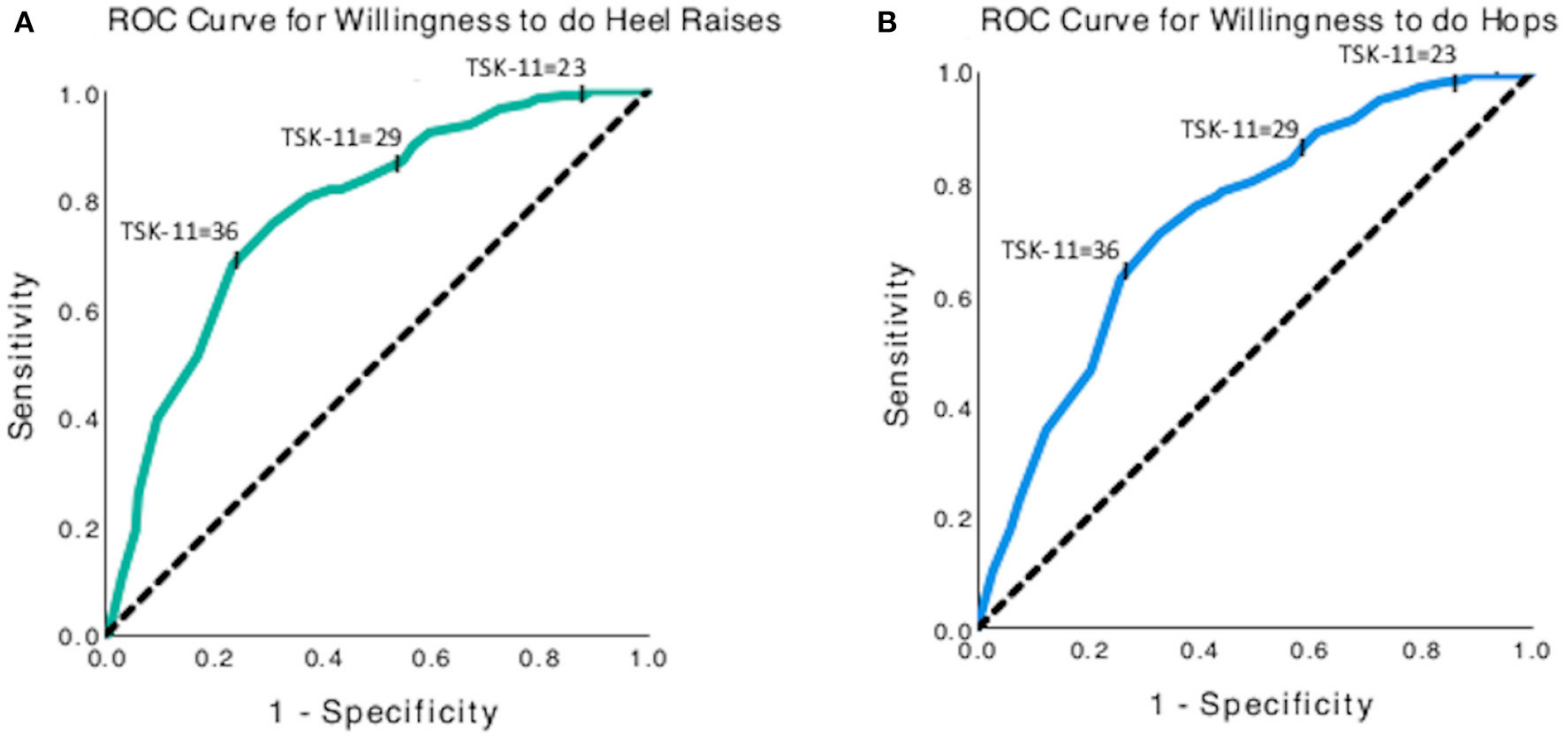
Receiver operating curves for using the TSK-11 score to predict willingness to complete **(A)** single leg heel raises, and **(B)** single leg hops. The y-axis represents sensitivity, and the x-axis represents (1-specificity).

The total score on the TSK-11 had a 77.6% (Area under the curve (AUC) 95% CI: 0.731–0.821, *p* < 0.001) chance of distinguishing between those who were willing and those who were not willing to complete a single leg heel raise (Figure 6A). The threshold of 23 for Low kinesiophobia had high sensitivity (99.0%) for detecting respondents who may not complete the heel raises yet had low specificity (15.9%). The threshold of 29 for Moderate kinesiophobia had good sensitivity (90.0%) and poor specificity (43.3%) for detecting respondents who may not complete the heel raises. The threshold of 36 for High kinesiophobia had poor sensitivity (67.9%) and moderate specificity (76.6%) for detecting respondents who may not complete the heel raises. Similar patterns of sensitivity and specificity for kinesiophobia thresholds were determined for willingness to complete hops with an AUC of 0.73 (95% CI: 0.68–0.78, *p* < 0.001, Figure 6B).

**Figure 7:**
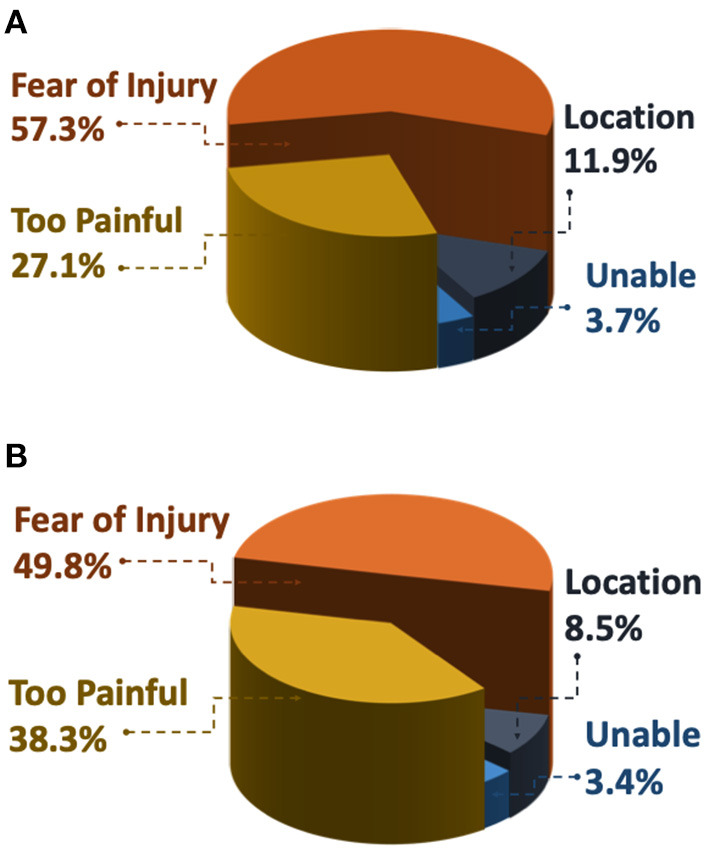
Frequency of reasons why respondents were not willing to do **(A)** three single leg heel raises and/or **(B)** three single leg hops. Respondents who choose not to perform the activity were then asked to select why from the following options: Fear of Injury: “I'm afraid I would hurt myself if I did this exercise,” Too Painful: “It would be too painful to do this exercise,” Location: “I am not in a location where I can try this exercise,” Unable: “I am unable to do this exercise on my painful side.”

Fewer respondents in the High-kinesiophobia group were willing to complete activities (24.4% of subgroup completed heel raises and 25.0% completed hops) than the other three groups, which ranged from 52.9 to 93.2% (*p* < 0.001 for all comparisons, Table 2). Among participants who declined to do the heel raises and the hops, the most common reasons for refusal was fear of injury (heel raise, 57.3%; hops, 49.8%) and that the activity was too painful (heel raise, 27.1%; hops, 38.3%; Figure 7).

### Minimal Clinically Important Differences for AT Symptoms

A pain MCID of 10 points out of 100 was chosen by nearly twice as many people (39.4% of respondents) as the larger MCIDs of 20 points (16.1%), 30 points (23.8%), and >30 points (20.4%, Table 4). The target percent decrease in pain increased by subgroup in parallel with larger MCIDs (10-point MCID = 25% [15.7–50.0]; 20-point MCID = 34.8% [30.8–50.3]; 30-point MCID = 47.6% [42.3–73.2], *p* < 0.001), yet there were no differences between pain-MCID subgroups in target pain intensity (Table 4). There was a statistically significant effect of group on TSK-11, but were no differences detected between pain-MCID subgroups (Table 4). The >30-point MCID subgroup had higher pain catastrophizing than the 10-point MCID subgroup (11.0[7.0–14.0] vs. 8.0[6.0–11.0], *p* = 0.001) and lower expected pain with heel raises than the 30-point MCID subgroup (Table 4).

**Table 4:** Based on response to minimal clinically important difference (MCID) questions for pain during single leg heel raises and duration of morning tendon stiffness, four subgroups were defined each symptom.

Movement-evoked pain MCID^*^	**10pt MCID**	**20pt MCID**	**30pt MCID**	**>30pt MCID**	***p*-Value**
	***n* = 174 (39.4%)**	***n* = 71 (16.1%)**	***n* = 105 (23.8%)**	***n* = 90 (20.4%)**	
**Expected pain with three heel raises** (0–100)	40.0 [19.8–63.3]	57.0 [39.0–65.0]	62.0 [40.5–71.0]	51.5 [32.8–64.0]	** <0.001**
*post-hoc*	0.220^a^	1.0^b^	**0.020** ^c^	1.0^d^	1.0^e^
**Target pain intensity** (0–100)	30.0 [9.8–53.3]	37.0 [19.0–45.0]	32.0 [10.5–41.0]	NA	1.0
**Target percent decrease** (%)	25.0% [15.7–50.0]	34.8% [30.8–50.3]	47.6% [42.3–73.2]	NA	** <0.001**
*post-hoc*	** <0.001** ^a^	** <0.001** ^b^	NA	NA	NA
**TSK-11** (11–44)	34.0 [26.8–37.0]	31.0 [27.0–38.0]	36.0 [33.0–38.0]	35.5 [28.0–39.0]	**0.020**
*post-hoc*	1.0^a^	0.280^b^	1.0^c^	1.0^d^	0.580^e^
**PCS-4** (0–16)	8.0 [6.0–11.0]	9.0 [6.0–12.0]	11.0 [9.0–12.0]	11.0 [7.0–14.0]	** <0.001**
*post-hoc*	1.0^a^	0.460^b^	1.0^c^	1.0^d^	**0.001** ^e^
Stiffness MCID^*^ **(min)**	**5 min MCID**	**10 min MCID**	**20 min MCID**	**>20 min MCID**	***p*** **-Value**
	***n*** **=** **153 (34.6%)**	***n*** **=** **70 (15.8%)**	***n*** **=** **120 (27.1%)**	***n*** **=** **96 (21.7%)**	
**Duration of morning tendon stiffness**	29.0 [15.0–59.5]	50.0 [30.0–67.0]	40.0 [29.0–60.0]	54.0 [25.0–63.0]	**0.020**
*post-hoc*	**0.020** ^a^	1.0^b^	1.0^c^	1.0^d^	**0.040** ^e^
**Target stiffness duration**	24.0 [10.0–54.5]	40.0 [20.0–57.0]	20.0 [9.0–40.0]	NA	**0.020**
*post-hoc*	**0.001** ^a^	** <0.001** ^b^	NA	NA	NA
**Target percent decrease**	17.0% [8.4–32.8]	20.0% [14.8–33.3]	50.0% [33.3–69.0]	NA	** <0.001**
*post-hoc*	0.160^a^	** <0.001** ^b^	NA	NA	NA
**TSK-11** (11–44)	34.0 [27.0–37.0]	35.0 [26.0–38.0]	35.0 [28.0–37.0]	37.0 [29.0–40.0]	0.200
**PCS-4** (0–16)	8.0 [6.0–11.0]	9.0 [6.0–12.0]	10.0 [7.3–12.0]	11.0 [8.0–14.0]	**0.007**
*post-hoc*	1.0^a^	1.0^b^	0.340^c^	0.580^d^	** <0.001** ^e^

Target percent change for pain was calculated as (MCID/expected pain)^*^ 100. Target percent change was calculated as (MCID/Duration of tendon stiffness)^*^ 100. P-values are Bonferroni adjusted for multiple comparisons. Data presented as median [interquartile range] with p-value for Kruskal Wallis test. All post-hoc tests were Mann-Whitney tests. NA, Not applicable because no acceptable MCID was identified for the largest MCID subgroup.

* *Two respondents chose not to report MCID for pain and three respondents chose not to report MCID for stiffness*.

a *P-value for comparison of Smallest MCID vs. Pain MCID of 20 pts/Stiffness MCID of 10 min*.

b *P-value for comparison of Pain MCID of 20 pts/Stiffness MCID of 10 min vs. Pain MCID of 30 pts/Stiffness MCID of 20 min*.

c *P-value for comparison of Pain MCID of 30 pts/Stiffness MCID of 20 min vs. Largest MCID*.

d *P-value for comparison of Pain MCID of 20 pts/Stiffness MCID of 10 min vs. Largest MCID*.

e *P-value for comparison of Smallest MCID vs. Largest MCID*.

*Statistically significant p-values are in bold*.

A 5-min stiffness MCID was the most common choice (34.6% of respondents) followed by a 20-min MCID (27.1%), >20-min MCID (21.7%), and 10-min MCID (15.8%). The 5-min MCID subgroup had a lower duration of tendon stiffness than the 10-min MCID subgroup (29.0 min [15.0–59.5] vs. 50.0 [30.0–67.0], *p* = 0.020). The other stiffness MCID subgroups had similar median duration of tendon stiffness ranging from 40.0 to 54.0 min (Table 4). The 5- and 10-min MCID subgroups had similar 17.0–20.0% targeted decrease in stiffness (*p* = 0.160). The 20- and >20-min MCID subgroups required at least a 50% decrease in duration of morning stiffness to be clinically meaningful (Table 4). There were no differences between stiffness MCID subgroups in TSK-11 (Table 4). The MCID >20 min subgroup had higher pain catastrophizing than MCID of 5 min (11.0 [8.0–14.0] vs. 8.0 [6.0–11.0], *p* < 0.001).

## Discussion

The purposes of this study were to (1) validate categories of fear of movement/re-injury on the TSK-11, and (2) develop patient-driven MCIDs for pain and stiffness. As hypothesized, subgroups with higher kinesiophobia had higher pain catastrophizing, higher Achilles tendon pain at rest, higher expected pain with activities, and a lower rate of willingness to complete Achilles tendon loading activities than subgroups with lower kinesiophobia. Moreover, these finding support the convergent validity of the TSK-11 categories with other abbreviated measures of fear of movement/re-injury and pain catastrophizing (12, 25). Yet there were no differences between kinesiophobia subgroups in movement-evoked pain intensity. For AT symptoms, the smallest MCID options, 10-point decrease in pain and 5-min decrease in tendon stiffness, were considered clinically meaningful by at least a third of the sample. The secondary purpose of this study was to determine whether fear of movement or pain catastrophizing were associated with the magnitude of the respondent's chosen MCID. These pain-related psychological variables were not associated with MCID in this sample.

The first hypothesis of the study was partially supported by an association between psychosocial factors (kinesiophobia, pain catastrophizing) and pain.

Consistent with previous literature, individuals with elevated pain catastrophizing often report higher pain intensity (26, 27). The new findings of this study are that psychosocial factors are also associated with other symptoms, including Achilles tendon stiffness. These findings indicate that higher kinesiophobia and pain catastrophizing are associated with more severe symptoms specific to the Achilles tendon region. In contrast to the study hypothesis, higher kinesiophobia was not associated with higher movement-evoked pain. Previous studies have indicated that the TSK-11 is positively correlated (*r* = 0.16–0.43) with average pain over the past 24 h to past week (9–11). One possible explanation of these contrasting findings is that the construct of “expected pain” in this study is similar to measures of recalled pain that correlate with kinesiophobia. In contrast, movement-evoked pain is a unique pain measure where the intensity is determined by a different combination of biological, motor, and psychological factors (28). Together these findings indicate that kinesiophobia likely contributes to individuals' willingness to complete an activity but not necessarily to their magnitude of pain with that activity. The fact that kinesiophobia is not simply a surrogate measure for movement-evoked pain intensity supports the clinical utility of the TSK-11.

The convergent validity of the TSK-11 was supported by groups with higher kinesiophobia being less willing to complete tendon-loading activities. This effect was most evident with the High kinesiophobia group, where only 24% were willing to complete three heel raises compared to 93% in the Minimal kinesiophobia group. These findings are consistent with Vlaeyen et al. (6), who reported that individuals with chronic low back pain and elevated kinesiophobia performed a lifting task for about half as long as individuals without elevated kinesiophobia. Therefore, high levels of kinesiophobia may interfere with participation in progressive tendon loading exercise programs (1), which commonly include heel raises and hopping, further indicating the need for clinical assessment of fear of movement. Interestingly, the findings of the current study differ from a recent study by Sigursdsson et al. (29), which reported that pain, rather than level of kinesiophobia, was associated with willingness to complete jumping activities in a laboratory setting. More quantitative and qualitative research is needed to understand how willingness to complete activities is affected by setting (at home vs. in a clinic), social support (alone vs. with a clinician), and pain severity.

The current study builds on previously published normative data to establish TSK-11 thresholds for low, moderate, and high kinesiophobia. This approach allows for interpretation of how the level of kinesiophobia in individuals with compare to those with other chronic musculoskeletal pain conditions. The kinesiophobia categories, 11–22 (minimal), 23–28 (low), 29–35 (moderate), 36–44 (high) on TSK-11 were supported by the high area under the curve in predicting willingness to do tendon loading activities of heel raises and hoping. The different thresholds indicate the tradeoff between sensitivity and specificity and provides enhanced clinical utility depending on the intended purpose of assessment. For example, use of the threshold of 23 with high sensitivity is most effective as a screening test, since a negative result (score <23) is useful in ruling out that the patient has high fear of movement. In contrast, the threshold of 36 with higher specificity is better as a confirmation test, where a positive result (score >36) is useful for ruling in fear of movement as a contributing factor to their willingness to do tendon loading exercises.

The kinesiophobia thresholds validated in this study, align with the quartiles of TSK-17 normative values from a patients seen at a pain management clinic and is similar to the TSK-11 threshold (>35) for high kinesiophobia in older adults with chronic pain (11, 18). Yet another study has proposed a dichotomous cut-off score of ≥17 as “high kinesiophobia” in teenagers following anterior cruciate ligament reconstruction (11, 30). Differences between studies using thresholds for “high kinesiophobia” likely depend on several factors about the population including age, injury chronicity, and injury type. The “high” kinesiophobia category validated in this study most aligns with thresholds determined from adults with chronic musculoskeletal pain (11, 18), and may not generalize to younger people with acute pain.

Approximately half of respondents indicated they would consider a 30% decrease in symptoms (pain MCID subgroups of 10 or 20 points and stiffness MCID subgroups of 5 or 10 min) as clinically meaningful, which is consistent with previous MCID recommendations (13, 14). A quarter of participants indicated that a 50% decrease in symptoms would be needed to be considered clinically meaningful (pain MCID subgroup of 30 points; stiffness MCID subgroup of 20 min). Together the majority of participants (nearly 80%) indicated that reducing their expected pain severity to the mild range (i.e., ≤ 40) would be clinically meaningful. Specifically, if all participants who indicated that a 10- to 30-point pain reduction would be clinically meaningful, this would place resulting median expected pain scores at 30–37 across the groups. These findings demonstrate that most persons with Achilles tendinopathy (AT) see meaningful clinical interventions as reducing their pain to a mild level, rather than eradicating pain completely. Respondents who selected the largest MCIDs had higher pain catastrophizing than respondents who selected the smallest MCIDs. For the subset of respondents who did not find any of the supplied MCIDs acceptable and may be seeking complete symptom resolution, pain education may be particularly impactful. Future research would need to test this hypothesis.

## Strengths and Limitations

A strength of this study is a large, international, community-based sample. Yet a limitation is that the inclusion criteria for AT relied on self-report and were not clinically evaluated specifically for this study. However, 94% participants reported seeing at least one provider for their AT symptoms, indicating that most respondents had been clinically diagnosed with AT. This sample also may not generalize to patients with mild AT symptoms; 74% of participants expected moderate to severe pain with heel raises (>30/100) and 80% had at least 20 min of morning stiffness, indicating that this sample included individuals with more severe or chronic AT (although chronicity of symptoms was not directly evaluated). Another limitation of self-reported outcomes is that the respondents' performance of the heel raise and hop tasks was not verified over video conferencing. It is possible that the study underestimated the number of people unwilling to tendon loading activities since performance was not visually confirmed.

Another strength of the study is utilizing the perspective of individuals with AT to determine the MCID, which is a limitation of other common MCID methods (31). In addition the cross-sectional design with respondents reporting their perception of what magnitude of change would be considered meaningful minimizes the effect of recall bias on the Global Rating of Change, for which other studies have compensated for by integrating the perspective of clinicians (31). Future work can further examine validity within a clinical trial to evaluate if the MCIDs for an anticipated change is equivalent to the MCID for a treatment-related change within a longitudinal study design. Another direction for future research is to submit the study protocol prior to commencing data collection to improve transparency (32).

## Conclusions

The convergent validity of TSK-11 subgroups in AT was supported by differences between groups in pain catastrophizing, pain at rest, expected pain with activity, and willingness to complete tendon-loading exercises. The prevalence of moderate to high kinesiophobia in this population (72% of this sample), combined with half of respondents being unwilling to complete tendon-loading exercises used in standard of care, underscore the importance of evaluating fear of movement, and pain catastrophizing in patients with Achilles tendon symptoms. The majority of participants (nearly 80%), indicated that reducing their pain severity to the mild range (i.e., ≤ 40) would be clinically meaningful, rather than eradicating symptoms completely. Respondents who selected the largest MCIDs had higher pain catastrophizing than respondents who selected the smallest MCIDs. To inform the focus of patient education, clinicians can screen for elevated kinesiophobia and pain catastrophizing that could hinder exercise participation and contribute to high expectations for symptom resolution.

## Data Availability Statement

The original contributions presented in the study are included in the article/Supplementary Material, further inquiries can be directed to the corresponding author/s.

## Ethics Statement

The studies involving human participants were reviewed and approved by University of Iowa Institutional Review Board University of Delaware Institutional Review Board. The patients/participants provided their written informed consent to participate in this study.

## Author Contributions

RC, ER, KGS, KAS, and GM: contributed to the concept, idea, and design of the study. RC, AP, and KH: contributed to the data collection and analysis. RC, ER, KAS, and GM: contributed to the fund procurement. RC, ER, and KGS: contributed in providing subjects. All authors contributed to the writing and consultation.

## Conflict of Interest

KGS receives speaker honoraria for talks on tendon injuries and for serving as an editor for Journal of Sports Physical Therapy. KAS serves as a consultant for Pfizer Consumer Health, Novartis Consumer Healthcare/GSK Consumer Healthcare, and receives royalties from IASP Press. GM receives royalties for key resources used for PNE (*Explain Pain, Explain Pain Handbook: Protectometer, Explain Pain Supercharged*, NOIgroup Publications, Adelaide, Australia), speaker fees for talks on contemporary pain education and has received support from: Reality Health, ConnectHealth UK, Seqirus, Kaiser Permanente, Workers' Compensation Boards in Australia, Europe and North America, AIA Australia, the International Olympic Committee, Port Adelaide Football Club, Arsenal Football Club. Professional and scientific bodies have reimbursed him for travel costs related to presentation of research on pain at scientific conferences/symposia. ER receives speaker fees for talks on tendinopathy and consults with various organizations on tendinopathy. The remaining authors declare that the research was conducted in the absence of any commercial or financial relationships that could be construed as a potential conflict of interest.

## Publisher's Note

All claims expressed in this article are solely those of the authors and do not necessarily represent those of their affiliated organizations, or those of the publisher, the editors and the reviewers. Any product that may be evaluated in this article, or claim that may be made by its manufacturer, is not guaranteed or endorsed by the publisher.

## Supplementary Material

Supplementary Video 1 Video of heel raise on left side.

Supplementary Video 2 Video of heel raise on right side.

Supplementary Video 3 Video of hop on left side.

Supplementary Video 4 Video of hop on right side.

Supplementary Table 1 Calculations for conversion of TSK-17 thresholds to TSK-11 thresholds.
